# Protection of Health Imagery by Region Based Lossless Reversible Watermarking Scheme

**DOI:** 10.1155/2015/489348

**Published:** 2015-11-15

**Authors:** R. Lakshmi Priya, V. Sadasivam

**Affiliations:** PSN College of Engineering and Technology, Melathediyoor, Tirunelveli 627152, India

## Abstract

Providing authentication and integrity in medical images is a problem and this work proposes a new blind fragile region based lossless reversible watermarking technique to improve trustworthiness of medical images. The proposed technique embeds the watermark using a reversible least significant bit embedding scheme. The scheme combines hashing, compression, and digital signature techniques to create a content dependent watermark making use of compressed region of interest (ROI) for recovery of ROI as reported in literature. The experiments were carried out to prove the performance of the scheme and its assessment reveals that ROI is extracted in an intact manner and PSNR values obtained lead to realization that the presented scheme offers greater protection for health imageries.

## 1. Introduction

Modernization in information and communication technologies has improved the quality of health care services being offered. Teleradiology is a technique in which patient images are transferred from one location to another for the sharing studies with other radiologists and physicians [1]. However the security of images transmitted is of great concern as it is also easy for hackers to modify or tamper medical images during exchange through insecure public networks. Therefore, it is crucial to offer protection for medical images to prevent any unauthorized modification or misuse and the security issues are viewed in three aspects, namely, confidentiality, authenticity, and integrity [2]. Confidentiality guarantees that only authorized users have access to the medical image, authenticity ensures that the images belong to the correct patient and are issued from correct source, and integrity certifies that the images have not been tampered by unauthorized person.

Digital watermarking is an accepted technique in the field of intellectual property and information security for both research and applications in the last decade. Watermarking modifies the pixel of image in an unnoticeable manner in order to insert a secret message within the image [3, 4]. Modification in medical images due to insertion of watermark is not accepted. The watermarking scheme is characterized by capacity, robustness, and invisibility apart from security requirement.

Capacity refers to the amount of information that can be embedded into the watermark which can be recovered without errors; robustness refers to the ability of the embedded message to overcome the insertion problem such as alteration in the pixel and information loss and invisibility refers to high fidelity, that is, original image, and watermarked image should sound similar.

Watermarking techniques for medical images have been developed in spatial domain as well as in frequency domain [5]. In spatial domain, data is embedded directly into the host image and in frequency domain data is embedded in transformed image.

Watermarking techniques are categorized as robust, fragile, and hybrid according to the purpose they serves. Robust watermarks are used for copy protection and copyright protection [6, 7] as they are resistant to image processing operations like rotation, scaling, cropping, and so forth. Robust watermarking methods assume the transmission channel as lossless and hence embedding is done in either lossless or lossy environment. As a result, these watermarks can be retrieved even after intentional or unintentional attack. On the other hand fragile watermark cannot resist attacks of any type. These watermarks easily get modified when host image is tampered. Hence it is mainly used for authentication and integrity verification [8, 9]. Hybrid watermark takes the advantage of both robust and fragile watermark and hence is used for both privacy control and integrity control [10].

Literature survey reveals that there are three solutions to the security problem. The first solution is reversible watermarking. Here once the watermark is retrieved, the reversibility property ensures that the watermark can be removed completely without affecting the quality of original image [11, 12]. The second solution is irreversible watermarking which is not acceptable in medical field due to the alteration caused to the original image as in lossy image compression. The third solution is region based watermarking technique which involves separating the original image into region of interest (ROI) and region of noninterest (RONI). Embedding of watermark is done in the RONI region which leaves the region of diagnosis intact [13].

This paper proposes a new fragile region based lossless reversible watermarking scheme which provides authenticity and integrity to the medical images exchanged even in an insecure channel.

The paper is organized as follows. In Section 2 the related works are given in detail.  Section 3 deals with the proposed framework. The experimental results are presented in Section 4. The scheme is validated through the discussion of results in Section 5 and Section 6 finally concludes this paper.

## 2. Related Work

Earlier many region based watermarking techniques were proposed which divide the medical image into ROI and RONI and leave the ROI as foolproof, thus enabling it to be used for diagnostic purpose. Some of the watermarking techniques are capable of hiding electronic health record (EHR) and many other techniques were capable of detection and recovery of tampered portions of the image [14].

Coatriex et al. [15] have presented a watermarking technique to protect the image by splitting the image into protection zone and insertion zone. On the ROI, three signatures, namely, hash, single parity, and linear block code, are calculated and embedded into RONI region. This method assumes that ROI consists of 75% of the image.

Wu et al. [16] have presented two methods for detecting unauthorized alteration in the image. The image is first divided into nonoverlapping blocks. In one method using modulo operation authentication message of the other block is embedded in each block. In the second method information about ROI is embedded in other blocks. In case of tamper, ROI can be extracted from other blocks. The drawback of these methods is that payload depends on the size of ROI.

Chiang et al. [17] proposed two block based methods where one method is based on symmetric key cryptography and the other is based on modified difference expansion. The first scheme can recover whole image whereas the second one can recover ROI of the image. The image is first decomposed into 4 × 4 block. Average of each block is calculated. Averages are concatenated and encrypted. Two-level discrete wavelet transform (DWT) is used to identify smooth blocks. The encrypted average values are embedded in the smooth blocks. From the watermarked image the encrypted average values are decrypted. Average values are calculated and compared to identify the tampered regions. The scheme achieves high capacity but the drawback is that the capacity depends on the number of smooth blocks.

Guo and Zhuang [18] have put forward region based lossless watermarking. Their scheme is capable of tamper detection. Region of embedding is chosen in such a way that it does not intersect ROI. Digital signature is calculated based on hash value. Watermark is generated by concatenating patient details and digital signature and then it is encrypted using Rivest Cipher 4 (RC4). Watermark is embedded using difference expansion technique in the region of embedding. This method achieves high embedding capacity with better visual quality. However this method is too complex and if the tamper occurs in region of embedding, the authentication data may be lost.

Al-Qershi and Khoo [19] developed a two-dimensional difference expansion technique to achieve high capacity. The image is divided into ROI, RONI, and border pixels. The payload consisting of hash of ROI, bits of ROI, and LSB of border pixels is embedded into RONI using two-dimensional difference expansion techniques. Then an embedding map is generated. This is embedded in the LSBs of border pixels. At the receiver side LSBs of border pixels are retrieved and embedding map is extracted. By using the embedded map, payload is extracted and then hash is calculated. If retrieved hash and newly computed hash match, then the image is said to be authentic. If the image is not authentic, then from the bits of ROI embedded, the tampered pixels are replaced. However the maximum ROI size chosen was only 12% of the image. This is very low when compared with many other schemes.

A hybrid watermarking method was proposed by Memon et al. [20] which generates a fragile watermark and a robust watermark. First the image is split into ROI and RONI region. Fragile watermark is embedded into the Least Significant Bit (LSB) of ROI and robust watermark is embedded into the embeddable blocks of RONI using integer wavelet transform. Using LSB substitution, location map is embedded in LL3 of each block. The ROI is combined with RONI to form the watermarked image. Upon reception the image is once again split into ROI and RONI. Fragile watermark is extracted from ROI and verified for tampering of image. Robust watermark extracted is used to check the authenticity of the image. Though the method is capable of detecting the presence of tampering it does not clearly specify how original ROI is recovered in case of ROI is being tampered with.

Kim et al. [21] have presented a watermarking scheme in which the image is divided into variable size block using quad tree decomposition. Average of each block is computed and used for recovery purpose. The drawback of this method is that if the block containing the recovery data is tampered, then the original image cannot be recovered.

Another ROI based watermarking technique was proposed by Coatrieux et al. [22] in which three watermarks are generated for different purposes. First two watermarks are used for tamper detection and localization. Third watermark is used to identify the nature of alteration. Like any other region based method this method also splits the image into ROI and RONI. Based on ROI, three signatures (H1, H2, and H3) are generated and embedded in the RONI. Upon extraction it first checks the signature H1. If H1 does not match it indicates that the image is tampered. Further H2 is used to localize the tampered region. Then H3 is used to conclude whether the modification is global or in some region only. H3 is used to conclude whether the modification is global or in some region only. In order to find the type of modification it uses multiclass SVM classifier to detect eight types of modification. The drawback with this method is that the method seems to be highly complex and may fail in case of unanticipated alterations.

Das and Kundu [23] have proposed a region based watermarking technique to solve various issues in medical information management. In this method, two watermarks are embedded in zeroth and first Least Significant Bit (LSB) planes. First watermark consists of encrypted EHR/DICOM metadata, indexing key words, doctor's identification code, ROI information, and the side information. Tamper detection is achieved by embedding second watermark consisting of the binary location map. This method achieves superior performance in terms of tamper localization capability, higher capacity, and imperceptibility. However watermark is embedded in two bit planes which may result in image degradation.

The above techniques make it clear that watermarking technique proposed should satisfy the following requirements:

 P1: reversibility,
 P2: tamper detection and localization,
 P3: fragility,
 P4: blind detection,
 P5: security,
 P6: intactness of ROI,
 P7: imperceptibility,
 P8: capacity.

The study of literature reveals that some of the previous works were modality specific, or less secure, or capacity limited or not able to detect tamper or cannot recover ROI completely. In order to overcome these drawbacks, in this work a new fragile reversible region based watermarking technique for medical image satisfying requirements P1–P8 has been employed. The proposed method is imperceptible with high capacity at the same time capable of tamper detection and recovery also.

## 3. Materials and Methods

The proposed scheme consists of two main procedures: (a) generation of watermark and insertion and (b) extraction of watermark and verification. It generates two fragile watermarks to be embedded in the image using spatial domain technique. The first watermark consists of authentication and recovery information which contains security element such as digital signature evaluating the reliability of the image. The second watermark is to achieve reversibility consisting of vertices of ROI and run length encoded location map matrix. Compression techniques are used to reduce the size of the watermarks and to reconstruct the original image without any loss. The methods used in the scheme and the procedure for watermark embedding and extraction are presented below.

### 3.1. Arithmetic Coding

In the proposed method arithmetic coding technique is applied to compress the ROI. Arithmetic coding is a lossless data compression technique which compresses ROI with high coding efficiency so that the compressed ROI can be used during recovery process to get back the original when the ROI is tampered during exchange. In general arithmetic coding considers list of symbols as one entity [24]. The number of bits used to encode each symbol varies as per the probability assigned to that symbol. Frequently occurring symbols are coded with few bits whereas those symbols that do not occur frequently are represented with more bits.

In the proposed technique the input to the coding algorithm is the vector representing the ROI and the statistical data about the appearance of each symbol in the given vector. The outcome is the compressed code representing the ROI:

(1) $$\begin{array}{c} code=Arithmetic\left(\;ROIvector,count\;\right). \end{array}$$

The code thus generated is one of the elements of the watermark.

### 3.2. SHA 256

SHA 256 is being used to achieve authentication [25]. SHA 256 algorithm accepts variable size message and produces fixed size message digest. In the proposed method the input to SHA 256 algorithm is 8 × 8 blocks. The pixels are arranged in the form of a string as

(2) $$\begin{array}{c} S_{x}=S_{1,1},S_{1,2},S_{1,3}\cdots S_{1,j}{,S}_{2,1}\cdots S_{i\times j}, \end{array}$$

where *i* × *j* is the binary value of each pixel. SHA 256 accepts the string and produces 64 characters digest for each block. This digest is used to compute the digital signature which forms a part of the watermark:

(3) $$\begin{array}{c} Digest=SHA\;256\left(\;S_{x}\;\right). \end{array}$$

### 3.3. Digital Signature (DS)

Digital signature is computed from message digest generated through SHA 256 algorithm. RSA (Rivest Shamir Adleman) algorithm is used to create the digital signature. Two different keys are used for encryption and decryption. Private key is known only by the sender and it is used for encryption and public key is known by everyone and is used for decryption. The advantage of using private key by the sender is that the receiver can be convinced that the message is from the sender and have arrived intact.

### 3.4. Embedding Scheme

The proposed scheme has been brought from the conventional LSB scheme [26] by introducing reversibility through the location map using exclusive or logic operation between the original data and watermark (*W*). The location map is created in the size of original image. Hence, it contains a slot for every pixel. While embedding the watermark, it is filled by XOR between the original data and embedded data. This location map is used for extraction. The location map entries can be selected from the four options. They are given in Table 1.

After the extraction of watermark, the location map is checked. If the location map value is 0, the same data is retained for extracted image; otherwise, complement of the extracted data will be filled for extracted image. Thereby, the proposed scheme brings the reversibility in conventional LSB scheme.

The functioning of the method is as follows. First a location map matrix is created in the size of *M* × *N* × *K*, where *M* refers to the number of rows in original image, *N* refers to the number of columns in original image, and *K* refers to number of planes. This location map matrix will be updated while embedding is being done.

**Step 1 .**

Read the pixel value of an original image (OI).

**Step 2 .**

Read the watermark (*W*). It can be either 0 or 1.

**Step 3 .**

Modify the 1st bit position of the pixel value by the secret data bits (*W*). This is the watermarked pixel (*P*_*W*_).

After the modification, the location map matrix will be updated by the results XOR operation as discussed in Table 1.

By repeating the same procedure for all the remaining pixels, based on the size of secret data, the watermarked image (*I*_*W*_) is generated.

### 3.5. Generation of Watermark and Insertion

**Step 1 .**

Select and extract ROI from original image (OI).

**Step 2 .**

Generate recovery data by compressing ROI using arithmetic coding (ROI_comp_):

(4) $$\begin{array}{c} {ROI}_{Comp}=Arithmetic\;\;Coding\left(\;ROI\;\right). \end{array}$$

**Step 3 .**

Divide ROI into 8 × 8 nonoverlapping blocks.

**Step 4 .**

For every block in ROI compute average (ROI_av1_).

**Step 5 .**

Compute digest (DIGEST_hash1_) using SHA 256 for every block (HASH_ROI1_).

**Step 6 .**

Generate digital signature (DS1) using a private key from DIGEST_hash1_

(5) $$\begin{array}{c} DS1=RSA\left(\;{Digest}_{hash1}\;\right). \end{array}$$

**Step 7 .**

Generate authentication data (Adata) using public key, ROI_av1_, and DS1:

(6) $$\begin{array}{c} Adata=\left(\;DS1⊕{ROI}_{av1}⊕public\;\;key\;\right). \end{array}$$

**Step 8 .**

Concatenate recovery data (obtained from Step 2) and authentication data obtained from Step 7 to form first watermark (*W*):

(7) $$\begin{array}{c} I_{W}=\left(\;{ROI}_{comp}⊕Adata\;\right). \end{array}$$

In order to achieve reversibility a second watermark is generated which is embedded in the 0th bit position of the watermarked image (*I*_*W*_). The second watermark consists of vertices of ROI and run length encoded location map matrix.

### 3.6. Watermark Extraction and Verification

The extraction process is done as follows.

**Step 1 .**

Read the received watermarked image *I*_*W*_ in decimal and convert into binary.

**Step 2 .**

Read the 0th bit positions of the watermarked pixel that gives the vertices of ROI and compressed location map.

**Step 3 .**

Expand the location map and fill the 1st bit position of the watermarked pixel as below.

Retain the same value, if the location map value for the position is 0. Otherwise, fill the position by the complement of extracted value.

**Step 4 .**

From the extracted watermark read the public key, ROI_av1_, DS1, and ROI_comp_.

**Step 5 .**

Find ROI region and split it into 8 × 8 nonoverlapping blocks.

**Step 6 .**

Compute digest (DIGEST_hash2_) from HASH_ROI2_ using SHA 256 algorithm.

**Step 7 .**

Find the digital signature (DS2) using the public key.

**Step 8 .**

Compare the DS1 and DS2; if they are identical, then it is concluded that there is no tampering in ROI of the medical image.

**Step 9 .**

If DS1 and DS2 do not match, then it is found that the ROI is tampered. Compute average (ROI_av2_) for every extracted block in the extracted image. Now compare ROI_av1_ and ROI_av2_.

(i) If the averages do not match, then identify the specific block as tampered.
(ii) Expand ROI_comp_.
(iii) Replace the tampered block with expanded block.

## 4. Experimental Results

**Figure 1:**
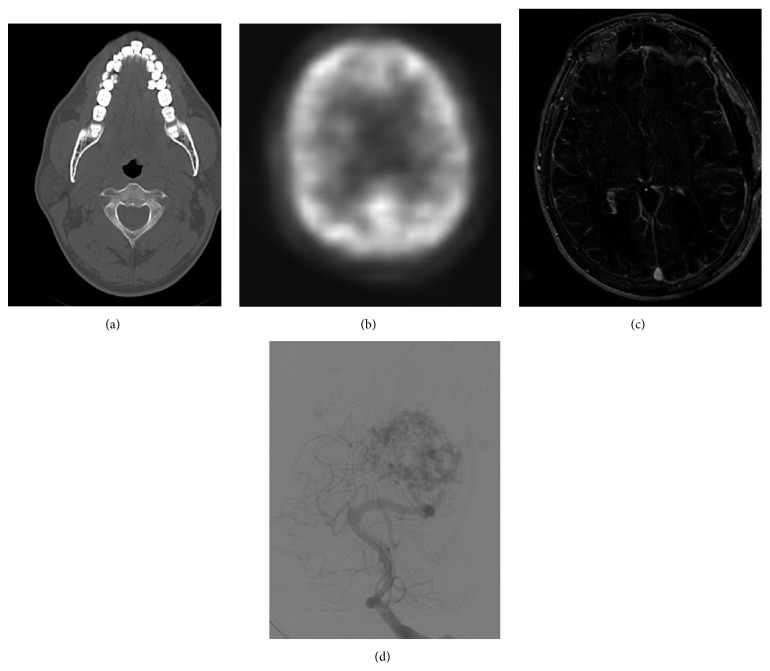
Sample medical images used in the experiment: (a) CT, (b) PET, (c) MR, and (d) XA.

National Electrical Manufacturers Association (NEMA) has made the use of digital imaging and communication in medicine (DICOM) standard as compulsory in Hospital Information Systems (HIS) and Picture Archiving and Communication Systems (PACS) to ensure compatibility among heterogeneous systems [27]. Hence the medical images used in this work were in DICOM format and are in four different modalities. The experiment was conducted on 25 medical images; however, for better presentation the results of four sample medical images are depicted in this work. Medical images are taken from OsiriX public database [28] and also obtained from physician.  Figure 1 shows some of the sample medical images used in the experiment. The test bed consists of

- MR scan image of resolution 512 × 512 pixels,
- XA scan image of resolution 1024 × 1024 pixels,
- CT scan image of resolution 512 × 512 pixels,
- PET scan image of resolution 128 × 128 pixels.

**Figure 2:**
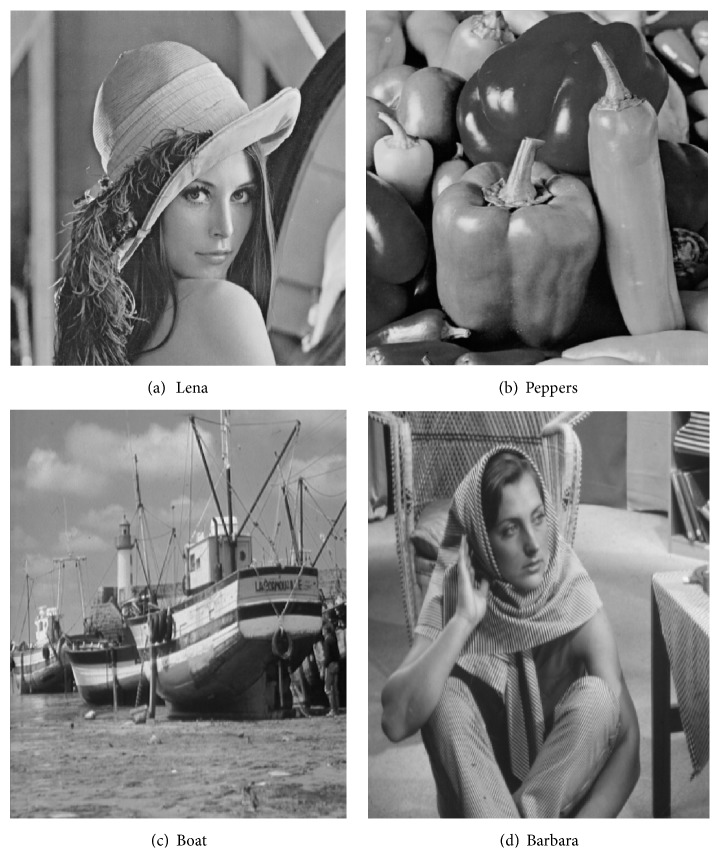
Sample standard images used for evaluation.

The second set consists of four grayscale standard images in bitmap format. These images are shown in Figure 2. These images were of size 512 × 512 (8 bits per pixel). The technique is implemented in MATLAB2011a and executed in Intel core i3 processor with 1 GB RAM.

The quantitative assessment on the quality of the watermarked images was calculated using Peak Signal to Noise Ratio (PSNR). PSNR provides the numerical measure of image distortion. It is defined as follows:

(8) $$\begin{array}{c} PSNR=10{log}_{10}\left[\;\frac{{\left(2^{p}-1\right)}^{2}}{MSE}\;\right], \end{array}$$

where *p* is number of bits per pixel. The higher the value of PSNR is, the lesser the image distortion is.

Mean square error (MSE) is given by

(9) $$\begin{array}{c} MSE=\frac{1}{M\times N}\overset{M-1}{\underset{i=0}{\sum }}\overset{N-1}{\underset{j=0}{\sum }}{\left(\;I-I_{W}\;\right)}^{2}, \end{array}$$

where *M* and *N* are the number of rows and columns of the image.

In the first experiment the relationship between selection of ROI on watermark size and imperceptibility in medical images was investigated. Though multiple ROI may also be chosen in this experiment only single ROI is chosen. The test is repeated for different sizes of ROI.  Table 2 shows the result obtained for PET image.

In the simulation, ROI is divided into 8 × 8 blocks and then the first watermark is generated after hashing and encryption. For example, consider the PET image of size 128 × 128 for which if the ROI chosen by the user was 64 × 82, then a digest was produced which is “*27369FC12E0D1C19 CD97DF16722BCDD5E4CE35B45BEF8A8C807A9E7EA22951EF.*” Digital signature algorithm was applied on the digest which produces the watermark of size 28586 bits. This watermark when embedded in PET image obtains the PSNR of 44 dB. Similarly the results obtained for MR, CT, and XA scan images are given in Tables 3–5.

The whole process was executed multiple times until the watermark size reaches 100000 bits or above except for PET image. Since the PET image is of size 128 × 128 pixels only, for that image, the experiment is executed for ROI of size 93 × 100 only which is 56% of the size of image. Average PSNR between the original and watermarked image was calculated. It is found to be 53.4 dB for MR scan image, 54.8 dB for CT scan image, 45.7 dB for PET scan image, and 62.3 dB for XA scan image.

**Figure 3:**
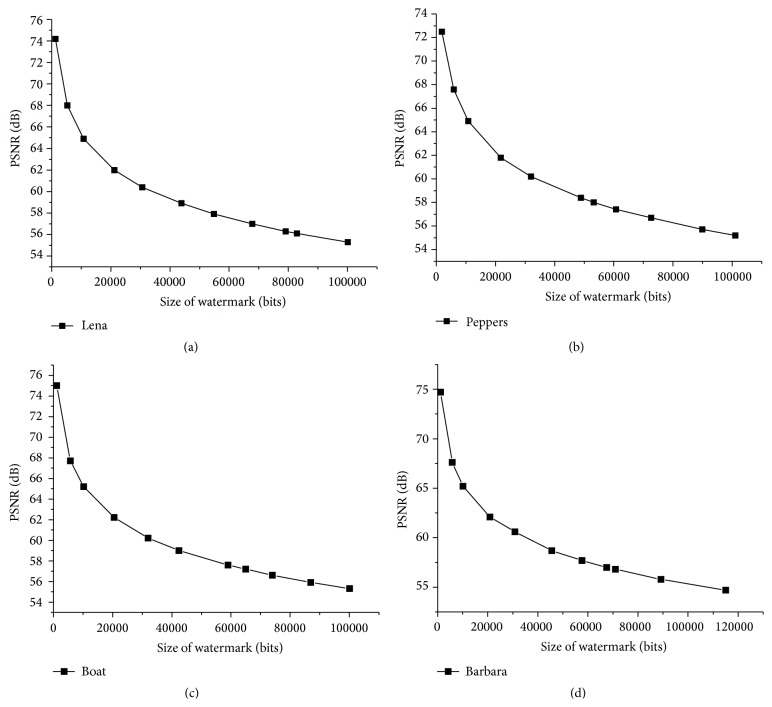
Performance in terms of watermark size versus PSNR: (a) Lena, (b) peppers, (c) boat, and (d) Barbara.

In the second experiment, standard images are used to test the performance of the proposed scheme.  Figure 3 shows the result of the proposed scheme in terms of size of watermark and PSNR. Since for each execution the ROI size may vary depending on the choice of the user, the size of watermark also varies.

Average PSNRs for standard images were calculated and it found to be 61 dB for Lena, 60.76 dB for peppers, 61.08 dB for boat, and 60.99 dB for Barbara image.

Run time complexity of the proposed system is studied by means of tic and toc commands in MATLAB. For the comparison of results in a fair way the ROI size is kept as 64 × 64 for all the images. It is found that the average time required for both embedding and extraction for PET image is 18/17 seconds, for MR image it is 8/8 seconds, for CT image it is 14/14 seconds, and for XA image it is 5/5 seconds.

## 5. Discussion

The suggested scheme makes use of two fragile watermarks. The first watermark consists of compressed ROI, digital signature, and average of block. This is used to verify the reliability of the medical image. Only with the first watermark reversibility cannot be achieved as the proposed method uses LSB embedding technique which is irreversible. Hence to achieve reversibility the need for second watermark arises which holds the location map matrix and vertices of ROI. Since the second watermark is used to achieve reversibility, during computation the length of second watermark is not included. Only the first watermark is used for computation of payload.

Reversibility is achieved in this proposed scheme by embedding the location map matrix in the image itself. It is completely blind, since it does not require either the original image or the original watermark for extraction. The projected scheme is moreover fragile in nature and hence any change in the watermarked image would affect the watermark hidden. In case of alteration the tamper detection and localization procedure would identify the corresponding tampered 8 × 8 block and mark it as tampered. However the method identifies only the tampered block and not the exact pixels. By reducing block size as 1 × 1 the method can identify the exact pixel. But here the block size is maintained as 8 × 8 in order to keep the run time minimum.

Security is achieved by digital signature algorithm because without proper key the verification cannot be performed. In the proposed method the first watermark is embedded in the second bit plane and the second watermark is embedded in the first bit plane. Even if the forger is capable of identifying the bit planes in which the first and the second watermarks are embedded, the forger cannot identify order in which the secret data is concatenated. If the forger modifies one or more bits, the extraction and verification algorithm exactly detects the tamper. Tamper localization procedure exactly spots the tampered block. Since compressed form of ROI is embedded in the image itself, ROI can be recovered without any loss and tampered block would be replaced with the recovered block. Thus the scheme achieves intactness of ROI making diagnosis error-free.

Table 2 gives the detailed experimental results including size of the PET image, size of ROI, watermark size, MSE, and PSNR for PET image. It can be observed from the results that when the size of the watermark increases the PSNR value drops indicating the inverse relationship between capacity and imperceptibility. According to Chen and Ramabadran [29] PSNR of above 40 dB is acceptable for medical images.  Table 3 contains the results obtained for MR scan image. PSNR of 48.1 dB is obtained for the watermark length of 103100 bits which is an acceptable value.

Table 4 gives the result for CT scan image. PSNR of 50.3 dB is obtained for the watermark size 101667 bits. Results of XA scan image are shown in Table 5. It is observed that PSNR of 57.3 dB is obtained for 106481 bits and it is also noticed that the size of ROI greatly influences the size of watermark payload.

From Tables 2–5 and Figure 3 it is evident that imperceptibility and watermark size (capacity) are in trade-off.  Figure 3 shows this trade-off relationship for general images.

The PSNR obtained for medical images is above 40 dB which is adequate for medical images. However, the PSNR for standard images drops below 60 dB for the watermark length of above 30000 bits. Therefore 30000 bits shall be the maximum size of watermark which will not introduce any visual artifacts for standard images. A comparison between the current work and reviewed works is presented in Table 6.

The limitation of this method is that it can only embed in sequential locations and not in random locations. Further this method assumes that ROI is selected by the radiologist to improve the security. Actually in real scenario, it is not confirmed that radiologist selects the ROI. Therefore either the radiologist must be made to select the ROI or its selection should be automated. More research is needed in this area as medical images are dissimilar in nature.

## 6. Conclusion

In this work, a new blind region based lossless watermarking technique with tamper detection and localization ability is proposed. It is applied to DICOM images of different modalities and also in general images. The content dependent watermark is generated dynamically based on the ROI selected. The process identifies tamper and effectively localizes it. The located tampered block is replaced by recovered block, thus maintaining the intactness of ROI. Further the scheme satisfies all requirements (P1–P8) as pointed out in Section 2. Moreover the PSNR values obtained are above 40 dB for medical images. In case of general images PSNR of above 60 dB is obtained for the size of the watermark lesser than 30000 bits. So this method offers better protection of health imagery compared to the other available techniques of this type.

## Figures and Tables

**Table 1:** XOR based matrix used in the proposed scheme.

Original data	Secret data	XOR result
0	0	0
0	1	1
1	0	1
1	1	0

**Table 2:** Embedding performance of PET image of size 128 × 128.

ROI size	Watermark size (bits)	MSE	PSNR (dB)
28 × 36	3923	0.445557	51.6
39 × 38	6040	0.688232	49.8
70 × 38	12749	1.503662	46.4
68 × 58	19770	1.929932	45.3
59 × 89	25870	2.374817	44.4
64 × 82	28586	2.571472	44
82 × 78	34770	2.908081	43.5
92 × 83	41314	2.964966	43.4
93 × 100	47339	3.190796	43.1

**Table 3:** Embedding performance of MR scan image of size 512 × 512.

ROI size	Watermark size (bits)	MSE	PSNR (dB)
36 × 38	2055	0.018265	65.5
76 × 101	14028	0.131912	56.9
91 × 124	20394	0.196762	55.2
105 × 151	30238	0.289795	53.5
131 × 170	44764	0.427246	51.8
143 × 188	52732	0.505875	51.1
158 × 202	63248	0.606247	50.3
155 × 238	74453	0.713287	49.6
195 × 262	103100	0.992874	48.1
228 × 269	125542	1.185028	47.4

**Table 4:** Embedding performance of CT scan image of size 512 × 512.

ROI size	Watermark size (bits)	MSE	PSNR (dB)
44 × 30	2455	0.019943	65.1
74 × 68	10581	0.074738	59.4
111 × 88	22833	0.154861	56.2
133 × 111	33163	0.210007	54.9
143 × 153	49651	0.330185	52.9
166 × 161	60479	0.371811	52.4
177 × 191	79364	0.488983	51.2
202 × 171	81431	0.489716	51.2
210 × 210	101667	0.603561	50.3

**Table 5:** Embedding performance of XA scan image of size 1024 × 1024.

ROI size	Watermark size (bits)	MSE	PSNR (dB)
48 × 32	2820	0.006744	69.8
60 × 57	4274	0.010193	68
105 × 81	12939	0.021969	64.7
117 × 93	13406	0.020687	64.9
201 × 129	36102	0.045143	61.5
153 × 180	42654	0.055649	60.6
195 × 195	56376	0.071297	59.6
236 × 208	69517	0.08065	59
198 × 252	74301	0.091957	58.4
240 × 306	106481	0.119026	57.3

**Table 6:** Performance comparison.

Scheme	ROI based	Block based	Identification of exact tampered block	Tamper recovery
Coatriex et al. [15]	Yes	No	Yes	No
Wu et al. [16]	Yes	Yes	No	With JPEG compressed form of ROI
Chiang et al. [17]	Yes	Yes	No	With average intensity of blocks
Guo and Zhuang [18]	Yes	Yes	Yes	No
Al-Qershi and Khoo [19]	Yes	Yes	No	With original pixels of blocks
Memon et al. [20]	Yes	Yes	No	No
Kim et al. [21]	No	Yes	No	With average intensity of blocks
Das and Kundu [23]	Yes	Yes	Yes	No
Proposed method	Yes	Yes	Yes	With compressed form of ROI
